# Effect of life skills building education and micronutrient supplements provided from preconception versus the standard of care on low birth weight births among adolescent and young Pakistani women (15–24 years): a prospective, population-based cluster-randomized trial

**DOI:** 10.1186/s12978-018-0545-0

**Published:** 2018-05-31

**Authors:** Jo-Anna B. Baxter, Yaqub Wasan, Sajid B. Soofi, Zamir Suhag, Zulfiqar A. Bhutta

**Affiliations:** 1 Centre for Global Child Health, The Hospital for Sick Children, Toronto, ON Canada; 2 Department of Nutritional Sciences, University of Toronto, Toronto, ON Canada; 3 Centre of Excellence in Women and Child Health, Aga Khan University, Stadium Road, Karachi, Pakistan

**Keywords:** Adolescence, Young adult, Nutrition, Micronutrients, Education, Preconception, Low birth weight, Pregnancy, Empowerment

## Abstract

**Background:**

Risk factors known to impact maternal and newborn nutrition and health can exist from adolescence. If an undernourished adolescent girl becomes pregnant, her own health and pregnancy are at an increased risk for adverse outcomes. Offering preconception care from adolescence could provide an opportunity for health and nutrition promotion to improve one’s own well-being, as well as future pregnancy outcomes and the health of the next generation.

**Methods:**

The Matiari emPowerment and Preconception Supplementation (MaPPS) Trial is a population-based two-arm, cluster-randomized, controlled trial of life skills building education and multiple micronutrient supplementation provided in a programmatic context to evaluate the impact on pre-identified nutrition and health outcomes among adolescent and young women (15–24 years) in Matiari district Pakistan, and the infants born to them within the context of the trial. The primary aim is to assess the effect of the intervention on the prevalence of low birth weight births (< 2500 g). The intervention includes bi-monthly life skills building education provided from preconception, and supplementation with multiple micronutrients during preconception (twice-weekly), pregnancy (daily), and post-partum (daily to 6 months). The standard of care includes non-regulated community-based health sessions and daily iron and folic acid supplementation during pregnancy. Additional outcome information will also be collected at set time periods. Among participants, these relate to nutrition (anthropometry, nutritional status), morbidity, and mortality. Among infants, these include birth outcomes (stillbirth, preterm birth, length of gestation, small for gestational age, birth defects), anthropometry, morbidity, and mortality.

**Discussion:**

Preconception care from adolescence that includes interventions targeting life skills development and nutrition is suggested to be important to improving the health and nutrition of adolescent and young women and their future offspring. This study is expected to offer insight into providing such an intervention both within a programmatic context and with an extended exposure period prior to conception.

**Trial registration:**

The MaPPS Trial was registered retrospectively on clinicaltrials.gov (Identifier: NCT03287882) on September 19, 2017.

**Electronic supplementary material:**

The online version of this article (10.1186/s12978-018-0545-0) contains supplementary material, which is available to authorized users.

## Plain English summary

Proper nutrition during pregnancy is important to the good health of mothers and their infants. However, many women in low- and middle-income countries who become pregnant have poor nutrition, and this can affect the outcome of their pregnancies and the health of their infants. If a well-nourished woman becomes pregnant, she is more likely to be healthy, have a healthy pregnancy, and give birth to a healthy infant. In this study, we aim to improve the nutrition and health of adolescent and young women (15–24 years) in rural Pakistan before they become pregnant, and so that they might be better nourished before becoming pregnant. To improve participants’ nutrition and health, we will provide life skills building education and multiple micronutrient supplements before they become pregnant and until 6 months after they give birth if they become pregnant. Because adolescent and young women in rural Pakistan are known to be undernourished and have babies early in life, they could benefit from this intervention. To determine the effect of the intervention, we will compare it to the care that women normally receive within the existing public health system. The main measurement that we will compare is how many infants are born with a birth weight that is low (< 2500 g), which suggests that the infants did not grow well during the pregnancy. Ultimately, we hope that the findings from this study will help us to understand what care should be given to women in places with poor nutrition before they become pregnant so that they have healthier pregnancies.

## Background

While the focus of global public health experts has been on the first 1000 days of life as the most developmentally critical period, risk factors known to impact maternal and newborn health can exist from adolescence [1]. If an adolescent girl becomes pregnant, her pregnancy is at an increased risk for adverse outcomes, including preterm birth and low birth weight (LBW) [2]. Furthermore, births to adolescent mothers can negatively affect both the mother and infant’s health in the future [3, 4]. As approximately 11% of all births are to adolescents 15–19 years of age, and more than 90% of these occur in low- and middle-income countries (LMICs), providing appropriate interventions prior to and during pregnancy will be crucial to reducing the burden of adverse outcomes [5].

Antenatal care (ANC) services offer an opportunity for maternal screening and intervention during pregnancy. However, in LMICs women tend not to report for care until the second trimester [6], thus the initial 100–150 of the first 1000 days are frequently missed. This presents a major limitation to ensuring adequate uptake of interventions for a reasonable duration, even though factors like nutritional status are crucial from the time of conception for placentation, organogenesis, prevention of congenital birth defects, and fetal growth [7–9]. Given the risks associated with anemia during pregnancy, iron and folic acid (IFA) supplementation is the standard of care during ANC [10]. Many experts have come to suggest that multiple micronutrient (MMN) supplementation should replace IFA given the prevalence of MMN deficiencies in LMICs, multiple causes of anemia, and synergistic interactions between micronutrients within biological processes [6, 11]. MMN supplementation during pregnancy can significantly decrease the risk of LBW births (relative risk: 0.88; 95% confidence interval: 0.85 to 0.91; high-quality evidence) compared to IFA [12], and among undernourished and anemic pregnant women MMN supplements could further improve infant survival and birth outcomes [13]. At this time, MMN supplementation has been neither accepted nor recommended by the World Health Organization [10].

Offering preconception care from adolescence could provide an opportunity for health and nutrition promotion to improve one’s own well-being, as well as future pregnancy outcomes and the health of the next generation [14]. Including a life skills building education (LSBE) component around empowering adolescent and young women to make informed health-related decisions will be key to sustainable and proficient uptake [14], as will be addressing underlying MMN insufficiencies [15]. Currently, there is a paucity of well-designed, interventional trials around the provision of MMN supplements preconceptionally. There are 4 existing trials which aim to investigate the efficacy of different preconception MMN supplements on birth outcomes within a trial setting (Table 1) [16–19]. Among the completed studies, the extent to which preconception MMN supplementation has affected each study’s respective primary outcome is variable.

**Table 1:** Summary of ongoing and completed interventional trials investigating preconceptional multiple micronutrient supplementation

Study name	Years active	Country	No. arms	Intervention	Minimum exposure to intervention
Potdar 2014 [16] (Mumbai Maternal Nutrition Project)	2006–2011	India	2	Arm 1: preconception: daily micronutrient-rich food snack; pregnancy: daily micronutrient rich food snack Arm 2: preconception: daily micronutrient-poor food snack; pregnancy: daily micronutrient poor food snack	3 months
Hambidge 2014 [17] (Women First Study)	2013– ongoing	multi-site (Congo, Guatemala, India, Pakistan)	3	Arm 1: preconception: daily MMN via lipid-based nutrient supplements (LNS) pregnancy: daily MMN via LNS Arm 2: preconception: none pregnancy: daily MMN via LNS Arm 3: preconception: none pregnancy: none	3 months
Owens 2015 [18]	2006–2008	The Gambia	2	Arm 1: preconception: daily UNIMMAP preparation tablet; pregnancy: daily IFA tablet Arm 2: preconception: placebo control; pregnancy: daily IFA tablet	not specified
Ramakrishnan2016 [19] (PRECONCEPT)	2011–2013	Vietnam	3	Arm 1: preconception: weekly MMN tablet; pregnancy: daily IFA tablet Arm 2: preconception: weekly IFA tablet; pregnancy: daily IFA tablet Arm 3: preconception: weekly FA tablet; pregnancy: daily IFA tablet	not specified

We hypothesize that the programmatic provision of LSBE and MMN supplements in a population-based setting in rural Pakistan will improve selected health, nutrition, and pregnancy-related outcomes among adolescent and young women 15–24 years old compared to those who receive the standard of care. For those who receive the intervention prior to and during pregnancy, we expect that there will be a decrease in the prevalence of LBW births. Conducting this trial within the context of the existing Pakistani public health program, we will assess the *effectiveness* of the intervention.

## Methods

The Matiari emPowerment and Preconception Supplementation (MaPPS) Trial is a two-arm, cluster-randomized, controlled trial of LSBE and MMN supplementation provided in a programmatic context to evaluate the impact on pre-identified nutrition and health outcomes among adolescent and young women, and the infants born to them within the context of the trial. Depending on whether participants become pregnant or not, there are three phases: preconception (maximum 24 months in duration); pregnancy (approximately 9 months in duration); and postpartum (maximum 12 months in duration). A detailed trial protocol has been developed in accordance with the SPIRIT guidelines [20] in a collaboration between the Aga Khan University (AKU; Karachi, Pakistan) and the Hospital for Sick Children (Toronto, Ontario, Canada). This paper addresses the methods for the pregnancy and postpartum phases of the MaPPS Trial, with the methods for ongoing measures taken throughout the preconception phase among participants who do not become pregnant appearing elsewhere [21].

The first participant was enrolled on 30 June 2017; enrolment is expected to be ongoing until July 2018; and planned data collection will continue until 2021.

### Objectives

The primary aim of the pregnancy and postpartum phases of the MaPPS Trial is to evaluate the impact of LSBE (provided bi-monthly) and supplementation with MMN (preconception: twice-weekly; pregnancy: daily) versus the standard of care (non-regulated community-based health sessions; preconception: no supplementation; pregnancy: daily IFA supplementation) on the prevalence of LBW births (< 2500 g). There are several secondary objectives for participants enrolled in the trial and the infants born to them to further determine the effect of LSBE and MMN supplementation compared to the standard of care. Among participants, these relate to nutrition (anthropometry [height, weight, middle upper arm circumference (MUAC)], nutritional status [iron, vitamin A, vitamin D]) and general health (e.g., morbidity, mortality). Among infants born to participants, secondary objectives include assessing the effect on birth outcomes (stillbirth, preterm birth, length of gestation, small for gestational age, birth defects), anthropometry (weight, length, MUAC, head circumference [HC]), morbidity, and mortality.

### Setting and participants

The MaPPS trial will be conducted in rural settings within Matiari district in Sindh province, Pakistan. Matiari is situated in the north-eastern part of Sindh, about 200 km away from Karachi, and representative of typical conditions in rural Pakistan. Including 1418 villages and a population of about 776,000, around 78,000 residents are adolescent and young women 15–24 years (based on regular surveillance data).

The nutritional status of Pakistani women is suggested to be suboptimal, and often attributed to that dietary staples are micronutrient poor [22, 23]. A comparable number of non-pregnant and pregnant women are anemic (51%) and experience iron deficiency anemia (20 and 26% among non-pregnant and pregnant women, respectively); deficiencies in other micronutrients are also common (e.g., vitamin A [43 and 49%] and vitamin D [85 and 86%]) [22]. Infants born to Pakistani women are consequently at an increased risk of poor growth and development. The national prevalence of LBW is 26%, yet this is higher among young mothers (29%) and in rural areas (33%) [23].

This trial is situated within Pakistan’s National Program for Family Planning and Primary Health Care as part of the Lady Health Worker (LHW) Programme. The LHW Programme aims to provide essential primary health services in the community using a cadre of female community health workers called LHWs [24]. Each LHW is affiliated with a health facility, where she reports to her lady health supervisor (LHS), and is responsible for approximately 1000 people in the community. LHWs visit the homes in their catchment area monthly and carry out community support sessions to disseminate health education messages [25]. The priority age groups for LHWs are children < 5 years, pregnant women, and couples eligible for family planning, thus the content of existing LHW educational materials primarily focuses on the health of married women and children [24].

### Eligibility criteria

As the MaPPS Trial is a population-based effectiveness study of LSBE and MMN supplementation from preconception, broad eligibility criteria for participation in the preconception phase were established to improve the generalizability of the trial findings. Specifically, this includes that the minimum age at enrolment is 15 years and the maximum age is 23 years, such that participants will not age out of the trial prior to conception; adolescent and young women must report to be physically able to comply with the trial intervention. Adolescent and young women are not eligible to enrol if they are currently pregnant (to be re-approached after giving birth), participating in a different nutrition trial, or intend to leave the trial area. They can be of any marital status.

To proceed to the pregnancy phase of the MaPPS Trial, participants will be included if they become pregnant within 24 months of recruitment. To ensure adequate exposure to the intervention, only women who become pregnant after 6 months of MMN supplementation will be formally considered a part of the per-protocol MaPPS pregnancy phase. To maintain the relationship with the local community, women in the intervention arm who become pregnant prior to 6 months of MMN supplementation will be retained in the trial and some routine data will be collected on them and their pregnancies. To further be included in the postpartum phase of the MaPPS Trial, a woman will have to have had a live birth.

### Design and sample size

**Fig. 1:**
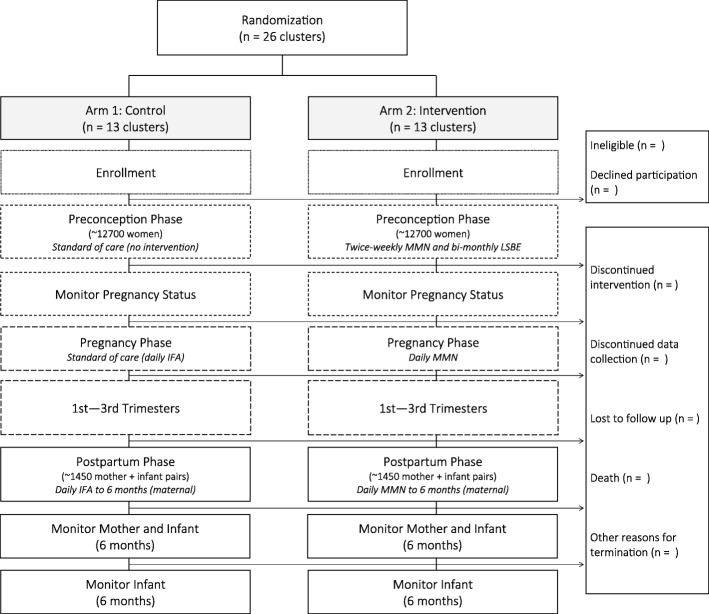
Matiari emPowerment and Preconception Supplementation (MaPPS) Trial flow diagram

The MaPPS Trial is a two-arm, parallel, prospective, cluster-randomized, controlled trial (Fig. 1). A cluster-randomized design was chosen to prevent contamination between the control and intervention arms through supplement sharing. The unit of randomization is previously defined and mapped health facility clusters, where health care services are provided to the surrounding population. A total of 26 clusters are available to be randomized (i.e., 13 clusters per arm). PASS 11 Software (NCSS, LLC., Kaysville, UT, USA) was used to determine all sample size calculations.

To observe a 25% relative reduction in LBW births (the a priori minimum detectable difference of public health importance for this trial) among pregnant women 15–24 years, assuming a 30% prevalence of LBW, icc = 0.011, k = 0.16, accounting for 10% attrition and probability of type I and type II errors of 0.05 and 0.20, respectively, the number births required per cluster was 56. This equates to 728 births per arm. To achieve these births, 12,712 women per arm will be required (total: 25,424 women).

### Cluster randomization and allocation concealment

The cluster allocation sequence was generated by an independent statistician (Simon Cousens, London School of Hygiene and Tropical Medicine) using a computer-generated stratification sequence in Stata software (StataCorp, College Station, TX, USA) based on the available health facilities in the district. The list was provided to the study manager, as full blinding is not possible given the nature of implementation of the intervention and control. Participants and LHWs are also not blinded given the differences between the intervention and control. The primary investigator, co-investigators, data collectors, phlebotomists, laboratory personnel, and data analysts are blinded to arm allocation. The allocation sequence will be provided to the trial Data Safety and Monitoring Board (DSMB) in cases where individual participants need to be unblinded given a suspected MMN supplement-related adverse events.

### Intervention and control

#### LSBE materials

In intervention clusters, enhanced LSBE materials have been developed on topics important to the empowerment of adolescent and young women to complement the existing LHW materials for use in community sessions. These will be implemented bi-monthly and attendance will be recorded. Three topic areas were prioritized: (a) delaying early marriage; (b) practicing appropriate personal and menstrual hygiene; and (c) the importance of good nutrition to health. Approximately 20 min is intended to be spent on each topic at community sessions. Integrated throughout the 3 topics are messages related to continuing one’s education, mental health (how to cope with stress, anxiety, and when you are upset), gender norms and equality, decision-making, advocacy, resiliency, participation, communication skills, facing challenges, agency, conflict resolution, and the prevention of violence.

Communication tools have been developed to assist the LHWs in conducting the LSBE-based community sessions. These were developed in coordination with Aahung, a Karachi-based non-governmental organization that aims to improve the sexual and reproductive health of girls (www.aahung.org; Karachi, Pakistan). Communication tools include a flipchart with 2 pictorials and discussion prompts per topic for use in sessions; and a brief summary pamphlet for distribution to participants. The flipcharts are similar to existing LHW tools for leading community sessions. A team of master trainers will be trained on all LSBE materials and appropriate communication techniques. The master trainers, in turn, provide comprehensive and interactive training for all intervention LHWs. Refresher training will also be provided on an ongoing basis throughout the duration of the trial.

#### Trial supplements

The MMN supplements used within the intervention arm of the MaPPS Trial are consistent with the UNICEF/WHO/UNU international multiple micronutrient preparation (UNIMMAP), and have been procured from the UNICEF Supply Catalogue (https://supply.unicef.org). Each tablet is 10 mm in diameter and includes vitamin A: 800 μg; vitamin D: 5 μg; vitamin E: 10 mg; folic acid: 400 μg; vitamin B1 (thiamine): 1.4 mg; vitamin B2 (riboflavin): 1.4 mg; vitamin B3 (niacin): 18 mg; vitamin B12: 2.6 μg; vitamin B6: 1.9 mg; vitamin C: 70 mg; iron: 30 mg; zinc: 15 mg; iodine: 150 μg; copper: 2000 μg; selenium 65 μg (Micronutrient Tabs, Pregnancy; Lomapharm, Emmerthal, Germany). All MMN supplements are maintained and stored by the study manager in a locked study office that is temperature controlled. A supply of MMN supplements is provided to LHWs monthly by a study monitoring team, which is unblinded to the allocation of the intervention and not involved in data collection. Once provided to participants, MMN supplements are stored in marked, opaque containers labelled with the participant’s ID and supplement batch number and expiry date.

The IFA supplements are provided to the control arm as part of Pakistan’s National Program for Family Planning and Primary Health Care free of charge [24]. Each tablet is required to include iron and folic acid amounts consistent with WHO recommendations (i.e., 60 mg of elemental iron and 400 μg of folic acid) [26].

Balanced-energy and protein (BEP) supplements will also be provided to those in the intervention and control arm if participants have a body mass index (BMI) < 18.5 kg/m^2^ during pregnancy, as per WHO recommendations [10]. Each sachet weighs 75 g and includes energy: 360 kcal; total fat: 24.8 g; carbohydrates: 19.5 g; protein: 9.9 g; sodium: 82.2 mg; potassium: 406.3 mg; vitamin A: 3.8 μg; vitamin C: 1.3 mg; calcium: 202.1 mg; iron: 0.87 mg (Afzaaish; Ismail Industries Ltd., Balochistan, Pakistan).

#### Supplement administration - intervention arm (MMN)

Upon enrolment in the trial, intervention arm LHWs will visit new participants within one month to provide the MMN supplements. A participant’s personal supply of MMN supplements will be monitored and replenished every month by her respective LHW throughout her enrolment in the trial. If a participant does not become pregnant within the context of the trial, she will take the MMN supplements for 24 months (the maximum duration of the preconception phase; majority of participants). If a participant becomes pregnant within the context of the trial, she will take the MMN supplements until 6 months postpartum.

During the preconception phase, participants are asked to consume 1 MMN tablet 2 days/week. To make the incorporation MMN supplementation into their routine easier, participants are requested to choose a consistent time on 2 days in the week that are separate from each other (e.g., Monday and Thursday, or Wednesday and Saturday) on which to take 1 MMN tablet with a glass of water and a meal. Each participant is provided with a 1 month supply of MMN supplements, plus a spare week in case of unscheduled missed visits. As such, participants receive 10 MMN tablets/month (4 weeks/month × 2 tablets/week + 2 extra tablets = 10 tablets total). During the pregnancy phase and for the first 6 months during the postpartum phase, participants are asked to consume 1 MMN tablet/day, given the higher daily micronutrient requirements [27]. As such, these participants received 38 MMN tablets/month (31 days/month + 7 extra tablets = 38 tablets total).

Participants are instructed that the MMN supplements are just for them and should be kept away from children. They are asked to ensure that the lid of the supplement bottle is tightly closed when not in use and stored away from light and humidity. It is also explained that some people experience mild side effects when they start taking the MMN supplements (e.g., nausea, vomiting, diarrhea, stomach pains), but that taking it with food should make side effects less likely to happen. If they miss a dose, participants are instructed that they should take 1 MMN tablet the next time that they remember, but never to consume more than 1 MMN tablet/day. If a participant has any side effects, is concerned, or has questions, she is instructed to contact her LHW and not study personnel since study personnel are blinded to whether participants are in the control or intervention arm. Participants are also asked not to take non-trial administered supplements while they are enrolled in the trial.

#### Supplement administration - control arm (IFA)

If a participant is in a control cluster, IFA supplements will be administered within 1 week of confirmation of a pregnancy. Daily IFA supplementation is part of the standard of care during pregnancy in Pakistan, thus LHWs are asked to continue their usual counselling. As the LHW program-supplied IFA supplements come in blister packs, blister packs will be cut up to aid monitoring so that participants receive 38 IFA tablets/month (31 days/month + 7 extra tablets = 38 tablets total), thus matching the method of provision for the MMN supplements. As in the intervention arm, a participant’s personal IFA supplement supply is monitored and refilled every month by her respective LHW. Daily IFA supplementation is to continue until 6 months postpartum.

#### Supplement administration – Both arms (BEP)

Participants in the intervention and control arm with a BMI < 18.5 kg/m^2^ will additionally be provided with daily BEP sachets for the duration of their pregnancy. Consistent with the other supplements consumed during pregnancy, BEP sachets will be provided and monitored monthly by LHWs (38 BEP sachets/month).

### Household listing

To identify young women who may be eligible to participate in the MaPPS Trial, a household-level listing of those living in LHW-covered areas of Matiari district was completed. The household listing step is consistent with AKU’s existing surveillance program within Matiari district, and took place from December 2016 – May 2017. It allowed for enumeration of the trial population and identification of young women who met the age criteria for enrolment. Adolescent and young women who met the age criteria were further asked questions to determine their potential eligibility (i.e., screening questions about their intention to remain in the trial area; involvement in other nutrition trials; and any complicated medical conditions that might prevent them from being able to take MMN tablets).

### Enrolment

Adolescent and young women identified in the household listing as provisionally eligible are further contacted by study data collectors at their homes to confirm their potential interest and eligibility. Provisionally eligible participants will be invited to participate in the preconception phase of MaPPS Trial in conjunction with their family members and/or husbands, and data collectors will explain the purpose and voluntary nature of the trial; participation components; and potential benefits and harms prior to obtaining written informed consent. If participants are < 16 years of age, assent will also be obtained as necessary. It is anticipated that enrolment will take at least 1 year.

Upon enrolment in the trial, a questionnaire designed to collect information on demographics; socioeconomic status (SES); reproductive health and history; life skills, empowerment, and social determinants of health (SDH)-related factors (Table 2); supplementation practices; and access to LHWs will be administered. The questionnaire has been adapted from the existing demographic health survey for Pakistan and several standardized assessment tools. All participants will also undergo anthropometric (height, weight, MUAC) and hemoglobin concentration measurement.

**Table 2:** Factors relating to life skills, empowerment, and SDH captured within trial questionnaires

Domains	Includes information pertaining to	Evaluation tool
Nutrition	Household food insecurity	Household Food Insecurity Access Scale [30]
Health	Eating habits Body image Physical activity	Health Behaviour in School-aged Children [31]
Perception of support	Family, peer, and school	Multidimensional Scale of Perceived Social Support [32]
Decision making	Family, food, health care, and daily needs	Pakistan Demographic Health Survey [23]
Psychosocial health	Self-efficacy Stress Mental health (depression, anxiety, and stress)	Generalized self-efficacy scale [33] Perceived stress scale [34] Depression, Anxiety, and Stress Scale-21 [35]
Violence	Intimate partner violence Exposure to violence	Conflict Tactics Scale [36] Pakistan Demographic Health Survey [23]

### Visit schedule

All visits with trial participants will occur at their homes. Each phase of the trial has a different number of visits, depending on how long the participant remains in the respective phase. Participants who do not become pregnant within the context of their enrolment will be followed maximally for 24 months in the preconception phase. The majority of participants are expected to fall into this category, and the ongoing measurements collected during this time are described in detail elsewhere [21].

Participants will be instructed about the appropriate signs to recognize pregnancy (e.g., amenorrhea), and amenorrhea will be assessed at monthly LHW visits. Women who become pregnant or suspect they may be pregnant will be asked to contact study personnel or their LHW, such that pregnancy confirmation might occur. Depending on when a participant becomes pregnant, her exact length of time in the preconception phase will vary. Upon confirmation of pregnancy, participants will be followed through to delivery. Participants and their family members will be asked to contact study personnel when labour begins. Furthermore, as LHWs will visit with participants at least monthly for the duration of the trial and study personnel will visit at set times, good rapport will be established between participants, their families and communities, LHWs, and study personnel, and all will be sensitized to notify of births as soon as possible. There are several additional measures to capture birth-related outcomes in a timely manner, including having personnel dedicated to keeping in phone contact with the participant close to her due date and employing a standby birth surveillance team, which is on call on holidays and weekends. Women who give birth to a live infant will proceed to the postpartum phase. Mothers and their infants will be followed until 1 year postpartum.

There will also be ongoing participant monitoring for the duration of the trial. Upon trial completion or withdrawal, participants will be asked to complete an exit questionnaire designed to assess their perception of their own health, the health of their infant, and satisfaction with trial participation.

#### Pregnancy phase

Given the frequent contact with and monitoring of participants, it is anticipated that most pregnancies will be recorded in the first trimester. Suspected pregnancies will be confirmed using a pregnancy test (HCG Rapid Test; ImuMed, Bammental, Germany) provided by a participant’s LHW. Confirmation of a pregnancy triggers completion of the first trimester assessment (Table 3). This includes formal collection of information on the participant’s last menstrual period, anthropometry (height, weight, MUAC), and mental health (Edinburgh Postnatal Depression Score [EPDS]) [28] by data collectors. All participants will also be asked to undergo a 5 mL blood draw. If a participant is found to have a BMI < 18.5 kg/m^2^, she will be provided with BEP supplements. Participants will be encouraged to go to the most convenient public sector health facility to obtain an ultrasound within 1 week of contact with data collectors. Among those who obtain ultrasounds, data will be collected on crown-to-rump length, estimated age, and general observations (fetal visibility, multiple pregnancy, heart activity, signs of abnormality). At 32 weeks of gestation, participants will undergo a repeat anthropometric (height, weight, MUAC) and mental health (EPDS) assessments.

**Table 3:** Enrolment and pregnancy phase data collection and measurement activities

Activity	Enrolment	Pregnancy	
	0 m	1st trimester	3rd trimester
Consent	X		
Demographics and SES	X		
Reproductive health and history	X		
Life skills, empowerment, and SDH	X		
Mental health	X	X	X
Anthropometry	X	X	X
Hemoglobin	X	X	
Serum ferritin	M	X	
Hepcidin	M	X	
Transferrin receptor	M	X	
Infection indicators (CRP, AGP)	M	X	
Vitamin A	M	X	
Vitamin D	M	X	
Pregnancy confirmation		X	
Ultrasound assessment		X	

X: all participants; M: micronutrient status subgroup participants only

##### Postpartum phase

Mothers and newborns will be visited within 24 h of birth to obtain anthropometric measurements of the newborn (weight, length, MUAC, HC), assess of the status of the newborn (e.g., birth defects), and interview the mother about the birth (Table 4). At 1 week postpartum, maternal status, complications during labour and delivery, and prelacteal and early breastfeeding practices will be assessed. Maternal anthropometric measurements (height, weight, MUAC) and a 5 mL blood draw will also be obtained. At 28 days postpartum, infant assessments will include morbidity and anthropometry (weight, length, MUAC, HC). Ongoing infant anthropometric and feeding assessments (breastfeeding and complementary feeding practices) will be conducted tri-monthly until the end of the trial (i.e., at 3, 6, 9, and 12 months). Maternal mental health (EPDS) and anthropometric assessments will be reassessed at 3 and 6 months postpartum, respectively.

**Table 4:** Postpartum phase data collection and measurement activities

Activity	Timing						
	24 h	1 w	1 m	3 m	6 m	9 m	12 m
Maternal							
Anthropometry		X			X		
Birth history		X					
Life skills, empowerment, and SDH							X
Mental health				X			
Hemoglobin		X					
Serum ferritin		X					
Hepcidin		X					
Transferrin receptor		X					
Infection indicators (CRP, AGP)		X					
Vitamin A		X					
Vitamin D		X					
Infant							
Anthropometry	X		X	X	X	X	X
Clinical assessment	X						
Feeding assessment	X			X	X	X	X
Morbidity			X				

X: all participants

#### LHW visits

Because LHWs are required to visit households in their catchment area monthly, these visits will serve to monitor menses, new marriages, and suspected pregnancies throughout the duration of the trial, in addition to their mandated function [24]. All trial-administered supplement provision and consumption will also be recorded at these visits.

#### Adherence, morbidity, and mortality monitoring

All participants will further be visited by an independent surveillance team to collect morbidity and self-reported adherence data every 3 months (Table 5). These visits will also allow for the observance and collection of data on non-trial administered nutritional supplement consumption.

**Table 5:** Monitoring data collection activities ongoing throughout the trial

Measurement	Monthly (LHW)	Quarterly (Monitoring Team)
Adherence by pill count^a^	i^b^	
Adherence by self-reported frequency^c^		i^b^
Side effects		X
Acceptability		i^b^
Morbidity (maternal and/or infant)		X
Mortality (maternal and/or infant)		X

X: all participants; i: intervention group only

^a^Adherence by pill count defined as number of tablets apparently consumed/number of possible supplements consumed given days enrolled in the study

^b^Among those women who become pregnant in the control group, measure will also be monitored at the indicated intervals from time of confirmation of pregnancy until 6 months postpartum

^c^Adherence options by self-report during preconception include twice weekly, intermittently, or not at all; during pregnancy and postpartum include daily, on alternate days, intermittently, or not at all

### Data collection

Data collection tools have been customized for each type of visit, and data is collected orally given the low literacy rates in the trial population. Trained study personnel will collect all trial outcome data using questionnaires, anthropometric measurements, and point-of-care tests. Standardized operating procedures have been developed for all measures in an effort to make data collection practices consistent. Extensive training sessions will be provided, employing classroom-based lectures, videos, hands-on practice, and mock interviews. As appropriate, training will be led by experienced personnel within the broader AKU network (e.g., data management unit, laboratory).

#### Questionnaires

For data collector-conducted visits with participants, tablets (Samsung Galaxy Tab. A T285; Samsung, Vietnam) will be used to collect questionnaire data. The tablets run a custom-made data collection application, which includes built-in logic and range checks, developed using Java (Oracle Corporation, Redwood Shores, CA, USA) and MySQL Lite (Oracle Corporation). LHW monitoring visits will use paper-based questionnaires given the large number of LHWs involved in the trial.

#### Anthropometric measurements

For participant measurements, height will be measured using a stadiometer (seca 213; seca, Hamburg, Germany); weight using a digital floor scale (seca 813); and MUAC using a measuring tape (seca 201). For infant measurements, length will be measured using an infantometer (seca 417), weight using an infant weigh scale (seca 354), and MUAC and HC using a measuring tape (seca 201 and 212, respectively). All measurements will be conducted in duplicate by two data collectors. In the case that two measurement exceed the allowable difference (length: < 1.0 cm; weight: < 0.5 kg; HC: < 0.5 cm; MUAC: < 0.5 cm), a third measurement will be obtained. The average (mean) of acceptable paired measures will be used in analysis. Standardized operating procedures for the anthropometric measurements were adapted from published reference materials [29].

#### Point-of-care hemoglobin assessment

To assess hemoglobin concentration, the HemoCue® Hb 301 System (HemoCue; Ängelholm, Sweden) will be used from blood collected via finger prick.

### Blood specimen collection and laboratory analyses

Trained phlebotomists (blinded to cluster allocation) will conduct all blood specimen collection using standard sampling procedures developed by the AKU Nutrition Research Lab (NRL). At each sampling time point, 5 mL of venous blood will be collected and stored in trace element-free vacutainer tubes (royal blue top; Greiner Bio-One, Monroe, NC, USA). Two drops of whole blood are immediately taken from the collection tube to assess hemoglobin concentration using the HemoCue® Hb 301 System point of care test. Because all blood specimens are collected at participants’ homes, they are then stored in a cool box with ice packs to maintain a temperature of 2–8 °C and protected from light exposure immediately after sample collection. Whole blood samples are received at the Matiari field site lab within 4 h, where the vacutainers are centrifuged to separate the serum from the red blood cells. The serum is pipetted into 1.8 mL cryovials (red top; Thermo Fisher Scientific, Waltham, MA, USA) and covered in aluminium to protect from light exposure. The cryovials are then stored between 2 and 8 °C, and transported in a cool box maintained at 2–8 °C to the AKU NRL once per week. Upon arrival at the AKU NRL, samples are analysed or stored in an ultra-low freezer maintained at − 70 °C for future use. Samples will be assessed for markers of nutritional status (iron [ferritin, transferrin receptor, hepcidin], vitamin A [retinol], and vitamin D [25(OH)D]) and inflammation (c-reactive protein, alpha-1-acid glycoprotein; Table 6).

**Table 6:** Assays and instruments used to determine constituents of interest from collected blood specimens

Micronutrient	Amount of serum (μL)	Assay method	Instrument used
Iron			
Ferritin	200	Immunoturbidimetric assay method	Cobas C311 Analyzer, Roche Diagnostics
Transferrin receptor	200	Immunoturbidimetric assay method	Cobas C311 Analyzer, Roche Diagnostics
Hepcidin	150	Enzyme-linked immunosorbent assay (ELISA)	Hepcidin-25, Bachem
Vitamin A (retinol)	200	Quantitative-high performance liquid chromatography photodiode array detection	Agilent HPLC, 1200/1260 Infinity Series with UV/PDA detection
Vitamin D (25(OH)D)	400	Electrochemiluminescence protein binding assay	Diasorin Analyzer, LIAISON
Inflammation			
C-reactive protein	200	Immunoturbidimetric assay method	Cobas C311 Analyzer, Roche Diagnostics
Alpha-1-acid glycoprotein	200	Immunoturbidimetric assay method	Cobas C311 Analyzer, Roche Diagnostics

### Data management

Data collection tablets are collected from study personnel on a daily basis so that questionnaire data can be uploaded to the AKU data management unit via a remote server (Windows Server 2008 R2; Microsoft, Redmond, WA, USA). Questionnaire data collected on paper-based forms are visually checked by field site supervisors for completeness before being sent to the AKU data management unit on a weekly basis for entry into a database. Double data entry is used to reduce data entry errors. The database was designed using MySQL software and entered using Visual FoxPro software (Microsoft). All collected data is kept under lock and key, and anonymized through the use of nine-digit participant identification codes. Data entered into the database is password protected.

### Outcome measures

To determine the prevalence of LBW at delivery, the primary outcome measure is birth weight < 2500 g. LBW will then be subdivided into very LBW (< 1500 g) and extremely LBW (< 1000 g). There are many additional secondary outcome measures within the trial related to nutrition and health that will be also assessed (see Additional file 1).

### Statistical analysis

For the primary outcome, the prevalence of LBW births will be compared by intervention arm, irrespective of maternal preconception supplementation duration or adherence (intention-to-treat). The per-protocol analysis will consider only those women with ≥6 months of exposure to the intervention. Because the prevalence of anemia and LBW are high in the trial population, an individual-level analysis using generalized estimating equations (GEE) will be employed to determine the estimate of the population average. Clustering will be accounted for within the GEE analysis. Sub-analyses will also be conducted, including determining whether there is a difference among those who received BEP and those who did not within and between arms, and whether there was a difference between adolescent and young women. There are several potential determinants which will be considered as covariates, such as age, parity, adherence, and SES. Relevant information will be collected at enrolment and throughout follow-up.

Summary estimates (e.g., means, proportions, counts) will be reported with 95% confidence intervals. Dichotomous outcomes will be compared using risk ratios with 95% confidence intervals, and the difference in continuous variables will be determined by comparing means. *P* values less than 0.05 will be considered statistically significant. Statistical analyses will be conducted using Stata software. The plan for the analysis of secondary objectives and outcome measures will be presented elsewhere.

### Study management

**Fig. 2:**
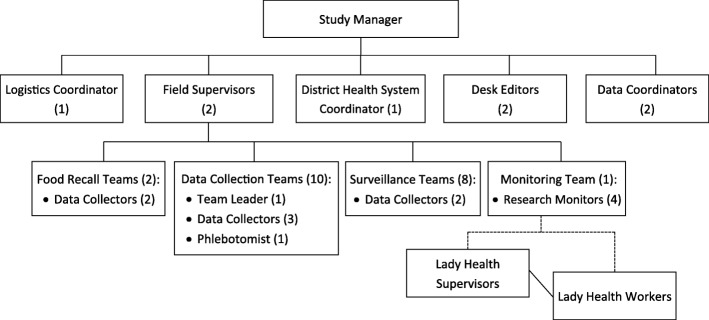
Organization of MaPPS Trial field research team

The field research team is overseen by the study manager, and includes several different positions (Fig. 2). Of note, data collection and food recall teams are responsible for collecting most primary and secondary outcome data; surveillance teams collect morbidity and supplement adherence data, as well as ongoing tracking of pregnancies and births; and monitoring teams are dedicated to coordinating with LHWs and conduct ongoing data collection quality checks. A DSMB has been developed including external international members with expertise in a wide array of related disciplines (pediatric and adolescent medicine, statistics, and nutrition). These individuals will provide expertise and recommendations relating to accumulated data about study operations and participant safety. When appropriate, they may be asked to make recommendations relating to continuation, modification, or termination of the trial. The DSMB meets with the study principal investigator and co-investigators to discuss progress on a quarterly basis.

## Discussion

Malnutrition prior to and during pregnancy can have important consequences for survival, acute and chronic disease incidence, healthy development, and economic productivity [8]. In this trial, we aim to assess the effectiveness of providing LSBE and MMN supplements to adolescent and young women in rural Pakistan from preconception, and compare this intervention with the standard of care on the prevalence of LBW births. Including a culturally-tailored educational piece aimed at empowering participants to make informed decisions about their health and well-being will be key to sustained behavioural change throughout the life-course. By implementing the intervention within the LHW Programme, we aim to gather important insight around how to achieve coverage within the existing programmatic context. This trial is distinctive in that it recruits both married and unmarried adolescent and young women. Consequently, we will be better able to ascertain the effect of the intervention on in the context of pregnancy, as well as investigate the effect on several domains of health and nutrition in the absence of pregnancy, as detailed elsewhere [21].

The WHO has not recommended antenatal MMN supplementation at this time [10]). The standard of care remains IFA supplementation, in spite of several large trials that show that MMN supplementation is efficacious in improving diverse health outcomes and meta-analyses that have found that MMN supplementation could be more effective than IFA in preventing adverse pregnancy-related health outcomes [12, 13]. Given that only around 50% of anemia is suggested to be caused by low iron stores, MMN supplementation could provide further benefit than IFA alone [11]. Furthermore, improving stores from preconception could be of added benefit because adolescent and young women would enter pregnancy with sufficient pregnancy stores.

Overall, the MaPPS Trial is expected to offer insight into providing an intervention that includes both LSBE and MMN supplementation to adolescent and young women from preconception and for a sufficient duration of time, and on the impact on their infants. So as to better understand the long-term effect of the intervention on development (e.g., growth and neurodevelopment) and diseases outcomes among infants born to study participants, we anticipate conducting further follow up studies in the future.

### Trial status

As of May 2018, participants are still being enrolled in the trial.

## Additional file

Additional file 1: Additional tables detailing secondary outcome measures and cut-points. Description of data: The additional file includes 3 tables detailing secondary outcome measures for participants and their infants. (DOCX 24 kb)

## Abbreviations

AKU: Aga Khan University
ANC: Antenatal care
BEP: Balanced energy and protein
BMI: Body mass index
DSMB: Data safety monitoring board
GEE: Generalized estimating eqs.
HC: Head circumference
IFA: Iron and folic acid
LBW: Low birth weight
LHS: Lady health supervisor
LHW: Lady health worker
LMIC: Low- and middle-income country
LNS: Lipid-based nutrient supplements
MaPPS Trial: Matiari emPowerment and Preconception Supplementation Trial
MMN: Multiple micronutrient
MUAC: Middle-upper arm circumference
NRL: Nutrition Research Lab
UNIMMAP: UNICEF/ WHO/UNU international multiple micronutrient preparation
WHO: World Health Organization
