# Supplementary material for: Effect of life skills building education and micronutrient supplements provided from preconception versus the standard of care on low birth weight births among adolescent and young Pakistani women (15–24 years): a prospective, population-based cluster-randomized trial
Source: Reprod Health. 2018 May 31;15:104. doi: 10.1186/s12978-018-0545-0 (PMC5984313; doi:10.1186/s12978-018-0545-0)
Supplement: Supplementary file 1 — Additional tables detailing secondary outcome measures and cut-points. Description of data: The additional file includes 3 tables detailing secondary outcome measures for participants and their infants. (DOCX 24 kb) [file 12978_2018_545_MOESM1_ESM.docx]

**Additional File 1 – MaPPS LBW Protocol Manuscript**

Additional Table 1. Summary of maternal nutritional status-related secondary outcome measures

| **Outcome** | **Type of variable** | **Variable definition** | **Analysis metric** | **Method of aggregation** |
| --- | --- | --- | --- | --- |
| Iron status | Efficacy | Serum ferritin concentration <15 µg/L (non-pregnant) or <12 µg/L (pregnant) | Value at specified intervals | 1) Mean  2) % above or below reference range |
|  | Efficacy | Serum transferrin receptor concentration >4.4 mg/L | Value at specified intervals | 1) Mean  2) % above or below reference range |
|  | Efficacy | Hepcidin concentrations <2.0 ng/mL | Value at specified intervals | 1) Mean  2) % above or below reference range |
| Vitamin A status | Efficacy | Serum retinol concentration <0.70 μmol/l (VAD) or <0.35 μmol/l (severe VAD) | Value at specified intervals | 1) Mean  2) % above or below reference range |
| Vitamin D status | Efficacy | Serum 25(OH)D concentrations <50 nmol/L | Value at specified intervals | 1) Mean  2) % above or below reference ranges |
| Inflammation | Safety | Alpha-1-glycolytic protein concentrations >1.0 g/L | Value at specified intervals | 1) Mean  2) % above or below reference range |
|  | Safety | C-reactive protein concentrations >5.0 mg/L | Value at specified intervals | 1) Mean  2) % above or below reference range |

Additional Table 2. Summary of maternal anthropometric, morbidity, and morality-related secondary outcome measures

| **Outcome** | **Type of variable** | **Variable definition** | **Analysis metric** | **Method of aggregation** |
| --- | --- | --- | --- | --- |
| Maternal height | Efficacy | Height at set points in time | Value at specified intervals | 1) Mean  2) % below -2 SD^1^ (from z-score) |
| Maternal weight | Efficacy | Weight at set points in time | Value at specified intervals | 1) Mean |
| Maternal MUAC | Efficacy | MUAC at set points in time | Value at specified intervals | 1) Mean |
| Maternal BMI | Efficacy | Derived from height and weight measurements at set points in time | Value at specified intervals | 1) Mean  2) % below -2 SD^1^ (from z-score) |
| Maternal morbidity | Safety | Occurrence of specified conditions among women during pregnancy or within 42 days of termination of pregnancy | N/A | N/A |
| Maternal mortality | Safety | Death of a woman while pregnant or within 42 days of termination of pregnancy, irrespective of the duration and site of pregnancy, from any cause related to or aggravated by the pregnancy or its management but not from accidental or incidental causes | N/A | N/A |

^1^Only determined among adolescent participants (age <19 years) using the WHO growth standards

Additional Table 3. Summary of infant secondary outcome measures

| **Outcome** | **Type of variable** | **Variable definition** | **Analysis metric** | **Method of aggregation** |
| --- | --- | --- | --- | --- |
| Gestational age | Safety | Gestational age at birth (in days) | Value at birth based on LMP and ultrasound (when available) | Mean |
| Preterm birth | Safety | Birth occurring at <37 completed weeks or 259 days of gestation | Value at birth base on LMP and ultrasound (when available) | N/A |
| Stillbirth | Safety | A baby born with no signs of life (e.g., beating of the heart, pulsation of the umbilical cord or definite movement of voluntary muscles) at or after 28 weeks' gestation | N/A | N/A |
| Small for gestational age | Safety | Birth weight <10^th^ percentile for gestational age | Value at birth | N/A |
| Infant weight | Efficacy | Weight at set points in time | Value at specified intervals | 1) Mean  2) % below -2 SD^1^ (from z-score) |
| Infant length | Efficacy | Length at set points in time | Value at specified intervals | 1) Mean  2) % below -2 SD^1^ (from z-score) |
| Infant HC | Efficacy | HC at set points in time | Value at specified intervals | 1) Mean  2) % below -2 SD^1^ (from z-score) |
| Infant MUAC | Efficacy | MUAC at set points in time | Value at specified intervals | 1) Mean  2) % below -2 SD^1^ (from z-score) |
| Birth defects | Safety | External abnormalities, as determined by a trained professional | Observation at birth | N/A |
| Neonatal morbidity | Safety | Morbidity occurring between days 0-28 of postnatal life | N/A | N/A |
| Neonatal mortality | Safety | Death of live born infant within first 28 days of life; subdivided into early neonatal mortality (deaths between 0-7 days) and late neonatal mortality (deaths between 8-28 days) | N/A | N/A |
| Infant morbidity | Safety | Morbidity occurring after 28 days of postnatal life and before one year | N/A | N/A |
| Infant mortality | Safety | Death occurring after 28 days of postnatal life and before one year | N/A | N/A |

^1^Infant anthropometric measures for weight, length, and MUAC will be used to derive a length-for-age z-score, weight-for-age z-score, and MUAC-for-age z-score, respectively. Anthropometric measurements at each visit will be based on the mean of paired measurements.
